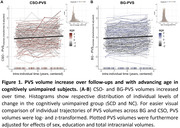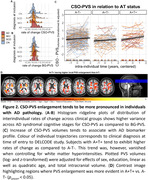# Perivascular space enlargement and cerebrovascular and Alzheimer’s diseases

**DOI:** 10.1002/alz.087129

**Published:** 2025-01-09

**Authors:** Jose Bernal Moyano, Inga Menze, Renat Yakupov, Pinar Kaya, Cagla Aki, Malte Pfister, Jonas Geisendörfer, Oliver Peters, Josef Priller, Anja Schneider, Klaus Fließbach, Jens Wiltfang, Katharina Buerger, Robert Perneczky, Stefan Teipel, Christoph Laske, Annika Spottke, Michael T. Heneka, Michael Wagner, Alfredo Ramirez, Steffen Wolfsgruber, Luca Kleineidam, Frank Jessen, Stefanie Schreiber, Emrah Düzel, Gabriel Ziegler

**Affiliations:** ^1^ German Center for Neurodegenerative Diseases (DZNE), Magdeburg Germany; ^2^ Institute of Cognitive Neurology and Dementia Research (IKND), Otto‐von‐Guericke University, Magdeburg Germany; ^3^ Centre for Clinical Brain Sciences, The University of Edinburgh, Edinburgh, Scotland UK; ^4^ Department of Neurology, Otto‐von‐Guericke University, Magdeburg Germany; ^5^ Charité – Universitätsmedizin Berlin, corporate member of Freie Universität Berlin and Humboldt‐Universität zu Berlin – Institute of Psychiatry and Psychotherapy, Berlin Germany; ^6^ German Center for Neurodegenerative Diseases (DZNE), Berlin Germany; ^7^ Department of Psychiatry and Psychotherapy, Technical University of Munich, Munich Germany; ^8^ University of Edinburgh and UK DRI, Edinburgh UK; ^9^ Department of Psychiatry and Psychotherapy, Charité, Charitéplatz 1, Berlin Germany; ^10^ German Center for Neurodegenerative Diseases (DZNE), Bonn Germany; ^11^ Department of Neurodegeneration and Geriatric Psychiatry, University Hospital Bonn, Bonn Germany; ^12^ Department for Neurodegenerative Diseases and Geriatric Psychiatry, University Hospital Bonn, Bonn Germany; ^13^ German Centre for Neurodegenerative Diseases (DZNE), Bonn Germany; ^14^ German Center for Neurodegenerative Diseases (DZNE), Goettingen Germany; ^15^ Department of Psychiatry and Psychotherapy, University Medical Center, University of Goettingen, Goettingen Germany; ^16^ Neurosciences and Signaling Group, Institute of Biomedicine (iBiMED), Department of Medical Sciences, University of Aveiro, Aveiro Portugal; ^17^ Institute for Stroke and Dementia Research (ISD), University Hospital, LMU Munich, Munich Germany; ^18^ German Center for Neurodegenerative Diseases (DZNE), Munich Germany; ^19^ LMU University Hospital, Munich Germany; ^20^ Munich Cluster for Systems Neurology (SyNergy), Munich Germany; ^21^ Department of Psychiatry and Psychotherapy, Klinikum der Ludwig‐Maximilians Universität München, Munich Germany; ^22^ German Center for Neurodegenerative Diseases (DZNE), Rostock Germany; ^23^ Clinic for Psychosomatics and Psychotherapeutic Medicine, Rostock Germany; ^24^ German Center for Neurodegenerative Diseases (DZNE), Tuebingen Germany; ^25^ Section for Dementia Research, Hertie Institute for Clinical Brain Research and Department of Psychiatry and Psychotherapy, University of Tuebingen, Tuebingen Germany; ^26^ Department of Neurology, University of Bonn, Bonn Germany; ^27^ Luxembourg Centre for Systems Biomedicine (LCSB), University of Luxembourg, Luxembourg Luxembourg; ^28^ Division of Neurogenetics and Molecular Psychiatry, Department of Psychiatry and Psychotherapy, Faculty of Medicine and University Hospital Cologne, University of Cologne, Cologne Germany; ^29^ Department of Neurodegenerative Diseases and Geriatric Psychiatry, University of Bonn Medical Center, Bonn Germany; ^30^ Department of Psychiatry & Glenn Biggs Institute for Alzheimer’s and Neurodegenerative Diseases, San Antonio, TX USA; ^31^ Deutsches Zentrum für Neurodegenerative Erkrankungen e. V. (DZNE) Bonn, Bonn Germany; ^32^ Excellence Cluster on Cellular Stress Responses in Aging‐Associated Diseases (CECAD), University of Cologne, Cologne Germany; ^33^ Excellence Cluster on Cellular Stress Responses in Aging‐Associated Diseases (CECAD), Faculty of Medicine and University Hospital Cologne, Cologne Germany; ^34^ Department of Psychiatry, University of Cologne, Medical Faculty, Cologne Germany; ^35^ Centre for Behavioural Brain Sciences (CBBS), Magdeburg, Sachsen‐Anhalt Germany; ^36^ Institute of Cognitive Neuroscience, University College London (UCL), London UK

## Abstract

**Background:**

Perivascular spaces (PVS) can become large enough to be visible in magnetic resonance imaging (MRI). The exact aetiology of PVS enlargement in humans remains, however, elusive and under continuous debate [1‐5]. Here, we tracked PVS volumes longitudinally over three years in 525 individuals along AD syndromal cognitive stages, namely cognitively unimpaired (CU), mild cognitive impairment (MCI), and Alzheimer’s disease (AD), to pinpoint conditions related to PVS enlargement.

**Method:**

We studied centrum semiovale (CSO) and basal ganglia (BG) PVS computationally over three to four annual visits in 525 DELCODE participants (CU/MCI/AD 417/72/36; 49.52% female, mean age 70.85 (SD 5.78)) [6]. We segmented PVS using a multimodal Hessian‐based filtering method [7] leveraging T1w and FLAIR imaging, which we validated against clinical visual ratings. We used linear mixed‐effect modelling to study temporal PVS volume changes. First, we tested whether PVS volumes increased over follow‐ups in CU. Second, we explored whether longitudinal PVS enlargement was associated across ROIs, and predicted by individual white matter hyperintensities (WMH), Amyloid and Tau positivity status at baseline in the entire cohort. We adjusted all analyses by age, sex, years of education, and total intracranial volume.

**Result:**

We observed PVS volume increase over follow‐ups in healthy ageing with a significant individual difference of change (Figure 1; BG: B=0.06 [95%‐CI 0.04‐0.08], p<0.001; CSO: B=0.06 [95%‐CI 0.04‐0.09], p<0.001). PVS enlargement in BG was associated with that in CSO (ρ=0.17, p_FDR_<0.001). Participants with greater baseline WMH volumes tended to have faster BG‐PVS enlargement (ρ=0.05, p_FDR_=0.06). Participants with both Amyloid and Tau positive tended to have faster CSO‐PVS enlargement than those with neither (Figure 2; Χ²(2)=5.07, p=0.079, η²=0.014).

**Conclusion:**

Given our findings, ageing is a primary driver of PVS enlargement. Associations between PVS and WMH underline shared cerebrovascular mechanisms. Detrimental cycles driven by neurotoxic waste accumulation might also contribute to PVS enlargement. Further research is needed to disentangle pathological cascades, their concurrent dynamics, and their unique contribution to disease progression.

References

10.1016/j.neurobiolaging.2022.01.006

10.18632/oncotarget.17724

10.1002/ana.26475

10.1161/STROKEAHA.117.017526

10.1016/j.clineuro.2019.05.002

10.1186/s13195‐017‐0314‐2

10.1007/BFb0056195